# Prolactin: A Mammalian Stress Hormone and Its Role in Cutaneous Pathophysiology

**DOI:** 10.3390/ijms25137100

**Published:** 2024-06-28

**Authors:** Ewan A. Langan

**Affiliations:** 1 Department of Dermatology, University of Luebeck, Ratzeburger Allee 160, 23562 Luebeck, Germany; ewan.langan@uksh.de; 2 Dermatological Sciences, University of Manchester, Oxford Rd, Manchester M13 9PL, UK

**Keywords:** prolactin, endocrinology, skin, hair growth

## Abstract

The hormone prolactin (PRL) is best recognised for its indispensable role in mammalian biology, specifically the regulation of lactation. Bearing in mind that the mammary gland is a modified sweat gland, it is perhaps unsurprising to discover that PRL also plays a significant role in cutaneous biology and is implicated in the pathogenesis of a range of skin diseases, often those reportedly triggered and/or exacerbated by psychological stress. Given that PRL has been implicated in over 300 biological processes, spanning reproduction and hair growth and thermo- to immunoregulation, a comprehensive understanding of the relationship between PRL and the skin remains frustratingly elusive. In an historical curiosity, the first hint that PRL could affect skin biology came from the observation of seborrhoea in patients with post-encephalitic Parkinsonism as a result of another global pandemic, encephalitis lethargica, at the beginning of the last century. As PRL is now being postulated as a potential immunomodulator for COVID-19 infection, it is perhaps timeous to re-examine this pluripotent hormone with cytokine-like properties in the cutaneous context, drawing together our understanding of the role of PRL in skin disease to illustrate how targeting PRL-mediated signalling may represent a novel strategy to treat a range of skin diseases and hair disorders.

## 1. Introduction

Whilst the ability of psychological stress to either trigger or exacerbate a range of skin diseases and hair disorders is well recognised, a detailed understanding of the biology of the brain–skin axis remains elusive. To date, research has largely focused on the role of the pituitary precursor polypeptide pro-opiomelanocortin (POMC) as key player along the brain–skin axis [1,2,3,4,5,6,7]. Indeed, POMC undergoes cleavage to form several hormones, including adenocorticotrophic hormone (ACTH) which regulates glucocorticoid secretion from the adrenal glands as part of the classical physiological response to stress. Indeed, human skin and hair follicles express a peripheral equivalent of the hypothalamic–pituitary–adrenal axis, first muted as a “stress skin response system” over two decades ago [8,9]. Human hair follicles actually display a functional equivalent of the hypothalamic–pituitary–adrenal axis and can even synthesize cortisol [10,11].

**Figure 1:**
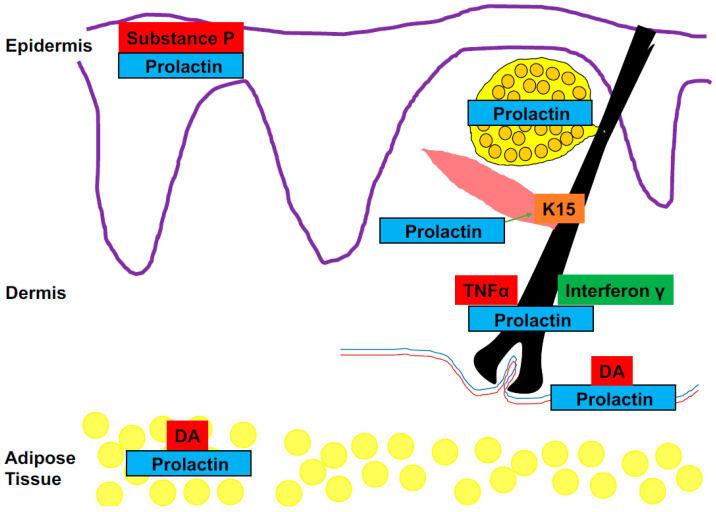
Site of prolactin (PRL) expression in the skin. PRL and/or PRL expression has been detected in the hair follicle, sebaceous gland, eccrine sweat gland, epidermis, adipose tissue and in lymphocytes. Expression is decreased by substance p in the epidermis and tumour necrosis factor alpha in the HF. In contrast, interferon gamma increases PRL expression in the HF (hair follicle). Whilst dopamine inhibits PRL release from the anterior pituitary and adipose tissue, this is not the case in the skin. Prolactin is novel regulator or keratin expression in the HF and HF stem cell biology (keratin 15 expression) in situ [17,18,19,20,21,22]. Abbreviations: DA—dopamine, K15—Keratin 15, TNFα—tumour necrosis factor alpha.

In fact, both the skin and hair follicles are important extra-pituitary sources of prolactin (PRL), a hormone which is best known for its central role in lactation (Figure 1) [12,13,14]. A role for PRL as a mediator along the brain–skin axis was first postulated over 30 years ago and the evidence was reviewed at the beginning of the last decade [15,16]. This review seeks to chart recent developments in our understanding of PRL in the neurocutaneous context and examine the extent to which modulation of intracutaneous and/or systemic PRL production may not only shed new light on the intricate regulation of the brain–skin axis but may also provide a novel therapeutic target for stress-mediated skin diseases (Table 1).

## 2. Background

The 23 kilodalton (kDa) hormone PRL is synthesised and secreted by the lactrotroph cells of the anterior pituitary gland. Unique amongst the hormones secreted by the anterior pituitary gland, its release is primarily under inhibitory control. The tuberoinfundibulum (TIDA) neurons of the arcuate nucleus secrete dopamine which binds to the dopamine 2 (D2) receptors on lactotrophes to inhibit PRL release. Whilst oestrogen and thyrotropin-releasing hormone may both stimulate PRL release, they likely do not play a central role in regulating pituitary PRL secretion in humans [48].

### 2.1. To What Extent Is PRL a “Stress Hormone?”

An important question when considering the role of PRL along the brain–skin axis concerns the extent to which PRL is a stress hormone [13,15]. The key protagonists of the hypothalamic–pituitary–adrenal axis, corticotropin-releasing hormone inducing adrenocorticotropic hormone secretion from the pituitary, which in turn triggers the production of glucocorticoids from the adrenal cortex, are immediately recognisable as stress hormones. Their crosstalk with the adreno-medullary system is central to mounting a life-preserving fight or flight response [49,50]. The crosstalk between PRL and the hypothalamic–pituitary–adrenal axis is less well understood.

However, several studies have drawn attention to the association between hyperprolactinaemia and increased adrenal steroid hormone secretion [51,52,53]. Patients with PRL-secreting pituitary adenomas have increased plasma concentrations of dehydroepiandrosterone sulphate (DHEAS) and whilst dopamine agonists reduce both serum PRL and DHEAS concentrations, they do not inhibit adrenocorticotropic releasing hormone secretion (ACTH) [54]. Taken together, these findings have led Lalli et al. [54] to propose that PRL is a “bona fide adrenocorticotropic hormone” synergising with ACTH in the endocrine control of adrenal cortex function via the steroidogenic transcription factor t (SF-1/NR5A1).

Furthermore, psychological stress is known to stimulate pituitary PRL secretion. Using the validated Trier social stress test [55], Lennartsson and Jonsdottir were able to demonstrate that acute psychosocial stress increased PRL release in both males and females under laboratory conditions [56]. Although the magnitude of the response exhibited wide inter-individual variation, the pattern did not differ between males and females. It should be noted that that several studies have failed to confirm the effect of stress on PRL release, or even shown a reduction [57,58]. Given that PRL is secreted in a diurnal pattern and exhibits sexual dimorphism, with baseline levels elevated in the early morning prior to waking and in females, experiments have to be carefully standardised. Given the range of physiological, pathological, genetic and medication-related factors that can influence serum PRL concentrations (Table 2), failure to control for these factors may partially explain discrepancies between studies examining the role of PRL in skin disease and hair disorders.

More recently, there has been renewed interest in the link between psychological stress and PRL release in the field of psychiatry. Traditionally, the hyperprolactinaemia seen in patients with major psychiatric illnesses, particularly schizophrenia and psychosis, was attributed to the anti-dopaminergic effect of anti-psychotic medication. Whilst hyperprolactinaemia is undoubtedly a side effect of anti-psychotic medication, albeit to varying extents depending on which dopamine receptors are predominantly antagonised, patients at high risk of developing psychosis, and and those with a first psychotic episode, exhibit increased serum PRL concentrations independent of anti-psychotic medication according to a recent meta-analysis [62,63]. In fact, the degree of hyperprolactinamia may negatively correlate with the severity of psychotic symptoms [64].

In addition to psychiatric illness, neurological conditions are also associated with hyperprolactinaemia. Most readily recognised is the PRL release following epileptic seizures, which may even help with differentiation between psychogenic non-epileptic and epileptic seizures [65,66]. A link between PRL and the skin was recently highlighted in a patient with thoracocervicofacial purpura and unwitnessed syncope, where hyperprolactinaemia helped establish the diagnosis of an epileptic seizure [67]. Hyperprolactinaemia has also been reported in neuro-inflammatory disorders, most notably in multiple sclerosis, and neurodegenerative diseases, including Parkinson’s disease [68,69,70,71]. Most significantly, the first indications that PRL may actually influence cutaneous physiology were generated from observations in patients with Parkinsonism with seborrhoea, including those with the post-encephalitic form following the last global pandemic [17,72,73]. Initially the evidence was indirect, often centred on the inhibitory effect of L-Dopa on sebum production [74].

However, direct evidence that PRL regulated aspects of sebaceous biology came from studies of ex vivo full-thickness skin organ culture. The addition of PRL increased sebaceous gland, and thus likely volume, mirrored by increases in the percentage of proliferating basal layer sebocytes in situ, based on the ratio between basal and mature sebocytes [75]. PRL also increased lipid production in a PRLR-signalling dependent manner. Namely, the addition of a pharmacological PRLR antagonist prevented increased lipid production. Finally, PRL also up-regulated 5-alpha-reductase type 1 protein expression in the human sebaceous gland ex vivo, suggesting some of its effect may occur via modification of peripheral androgen metabolism [75].

### 2.2. PRL in Cutaneous Physiology—Lessons from the Hair Follicle

Establishing the role PRL in HF biology was based on observations from ovine studies examining pelage and horn growth. Lincoln measured serum PRL concentrations at regular intervals over a period over 3 years in both domestic and wild sheep whilst recording wool (hair) growth [76]. In the mouflon sheep (*Ovis gmelini musimon*), the ancestor of domesticated sheep, serum PRL concentrations were intimately linked with pelage changes. Prolactin concentrations increased from winter to summer, which was followed by moulting and the development of a winter coat. In contrast, domesticated sheep had the highest PRL concentrations in the winter, and these were associated with continuous hair growth and the absence of a spring moult. The author suggested that the lack of seasonality in PRL secretion and hair growth may have been the result of selective breeding for continuous hair growth. These initial observations were confirmed in adult mouflon ewes, in which circannual patterns of PRL secretion were associated with seasonal hair growth and moult cycles, independent of temperature and photoperiod [77]. Murine studies, including those with PRL receptor (PRLR) knockout mice, have further substantiated the role of PRL in hair growth. Both PRL and the PRL identified in the HF in a hair cycle-dependent manner, with PRLR knockout mice having longer and coarser hair and PRL itself delaying anagen in vivo and promoting catagen in murine HF organ culture [78,79,80].

Although caution had to be exerted before extrapolating results from other species to human hair growth, especially given that human hair growth is non-synchronised and the evidence for seasonal growth patterns is limited [81,82], PRL has been confirmed as a potent regulator of human HF growth and cycling. Using microdissected human HF short-term organ culture, Foitzik et al. [83] could confirm HF compartment-specific and hair cycle-dependent expression of PRL and PRLR. Moreover, the addition of PRL resulted in hair shaft elongation and premature catagen development, evidenced by reduced proliferation and increased apoptosis of hair bulb keratinocytes. These data supported the earlier data in sheep and mice which demonstrated that the PRL directly influenced HF biology, rather than indirectly via modulation of other hormones. Furthermore, the data confirmed the skin as an extra-pituitary source of PRL, at the gene and protein level, which could modulate hair growth an autocrine and/or paracrine manner.

### 2.3. PRL and Alopecia Areata

Therefore, given that PRL modulated human HF biology, and bearing in mind its role as a stress hormone, it was conceivable that PRL played a role in stress-mediated hair diseases. Alopecia areata is a common, T-cell-mediated autoimmune disease which can be triggered and/or exacerbated by stress in patients with a genetic predisposition [84,85,86]. Follicular autoantigens, which are yet to be identified, are presented to autoreactive CD8+ T cells following the collapse of the HFs constitutive immune privilege. When this is accompanied by co-stimulatory factors during anagen (hair growth phase), including stress, the typical hair loss phenotype occurs.

Studies examining the role of PRL in alopecia areata are limited. Gönül et al. [87] found no difference in serum PRL levels between patients with alopecia areata and age- and sex- matched controls, although only one-third of patients had active disease at the time. El Tahlawi et al. [28] subsequently replicated this finding.

However, given that the skin and HFs are an extra-pituitary source of both PRL and PRLR, the authors went on to examine PRLR expression in scalp biopsies. Not only was the expression of the PRLR significantly increased in patients with alopecia areata, but its expression correlated with disease severity. The PRLR belongs to the type I cytokine receptor family. The PRLR dimerises following PRL binding and signals via the JAK2/STAT5, Ras-Raf-MAPK and PI3K/Akt/mTOR pathways. Interestingly, several JAK inhibitors, which are licensed for use in atopic dermatitis, have also shown promise in the treatment of alopecia areata [88]. It is at least conceivable that part of this response may be due to alterations in PRLR signalling. On a note of caution, PRLR signalling plays an important regulatory role in maintaining K15-positive epithelial stems cells in the HF, at least in situ. Therefore, blocking downstream PRLR signalling may have negative effects on HF cycling and growth [89,90]. Furthermore, as suggested by Gilhar et al. [89], alterations in IL-10 signalling as a consequence of JAK pathway inhibition may be undesirable given its role as the “guardian” of immune privilege.

Whether PRL and PRLR signalling play a significant role in the aetiopathogenesis of alopecia areata, potentially related to psychological stress, and to what extent this is due to systemic versus intracutaneous PRL production remain to be clarified. In addition, the role of PRL in follicular immune privilege, particularly in view of its stem cell regulatory role, deserve further attention and may ultimately help identify novel treatment approaches.

### 2.4. PRL and Autoimmune Blistering Disorders

In contrast to alopecia areata, where psychological stress may well play an aetiological role, the role of stress in the development of autoimmune blistering disorders is less well established. Apart from speculation that PRL may play a role in pemphigoid gestationalis, most evidence to date has centred on the PRL and pemphigus vulgaris [91]. Pemphigus vulgaris is an archetypical autoimmune disease characterised by the development of muco-cutaneous blister and erosions caused by the formation of autoantibodies targeting the dermosomal adhesion proteins desmoglein 3 and 1 may reportedly be triggered by stress in individuals with a genetic predisposition [92,93,94,95,96].

Exactly two decades ago, Khandpur and Reddy reported a case of pemphigus vulgaris in a female patient with hyperprolactinaemia [97]. Not only was there a temporal relationship between the clinical signs of hyperprolactinaemia (irregular menses, gynaecomastia, and galactorrhoea) and the development of muco-cutaneous widespread blistering, but treatment with bromocriptine, the potent dopamine agonist which suppresses PRL secretion, resulted in rapid resolution of the skin changes. Almost a decade later, Fallahzadeh et al. [98] confirmed significantly increased serum PRL levels in patients with pemphigus vulgaris when compared to age- and sex- matched controls. Although serum PRL concentrations did not correlate with disease site (mucosal and/or skin), they did significantly correlate with the extent of skin surface involvement. The finding of hyperprolactinaemia in pemphigus vulgaris has been replicated in several studies in up to 22% of patients, although a correlation between serum PRL and desmoglein 3 and 1 antibodies was not identified [36,37,38]. On the other hand, Lavaee et al. [99] did not find any significant difference between control subjects and patients with pemphigus vulgaris in females in terms of serum PRL concentrations. This may have been due to the small number of participants, which was further reduced when pre- and postmenopausal subjects were evaluated separately. This is important given that serum PRL levels vary between males and females and are influenced by menopausal status.

Prospective studies are required to definitively answer the question of whether PRL plays a role in the pathogenesis of pemphigus vulgaris given that PRL does play a role in other autoimmune diseases, at least partially via inhibition of negative selection of autoreactive B cells [100]. Furthermore, the effect of the anti-CD20 monoclonal antibody Rituximab on serum PRL concentrations may be worth evaluating, given that B cells express both PRL and the PRLR and that rituximab and corticosteroids were associated with a normalisation of serum PRL levels in a patient with seller granulomatosis mimicking a cabergoline resistant prolactinoma [101].

### 2.5. PRL and Lupus

Systemic lupus erythematosus is a chronic systemic autoimmune disease whose clinical manifestations are diverse and whose complex aetiopathogenesis remains incompletely understood [102]. Psychological stress is recognised as a potential contributory factor in the development of lupus but can also exacerbate the symptoms of the disease [103]. Moreover, the disease itself may be associated with neuropsychiatric symptoms and significant psychological morbidity [18,104]. In fact, psychological interventions can be utilised to improve disease activity and levels of stress, depression, and anxiety [105], as part of a multi-modal treatment strategy.

Given the female to male ratio of up to 9:1 in adult patients, the role of sex hormones in disease pathogenesis has long been speculated on [106]. Intriguingly, the first evidence of elevated serum PRL concentrations in systemic lupus was reported in a small study of eight male patients [19]. Furthermore, early trials of the dopaminergic agonist bromocriptine demonstrated a positive effect on disease control and a reduction in disease flares [107,108].

Over the next few decades, a range of studies were published with seemingly conflicting results. A number of studies confirmed hyperprolactinaemia in patients with lupus, where serum concentrations correlated with disease activity, serositis, and anaemia, and were even a potential biomarker of lupus nephritis, whereas other studies failed to replicate these associations [109,110,111,112,113,114]. Study size, design and power undoubtedly contributed to these divergent findings. However, two relatively recent meta-analyses have confirmed that systemic lupus is indeed associated with hyperprolactinaemia and that serum concentrations correlate with disease activity [115,116]. The prolactin gene −1149 G/T polymorphism has also been associated with lupus and rheumatoid arthritis, although this finding could not be replicated for the former in a meta-analysis [117].

Whilst it now seems clear that PRL is involved in the pathogenesis of lupus [118], via its participation in both the innate and acquired immune response, the exact role of PRL as an immune-stimulatory or immune-inhibitory factor requires careful dissection. Perhaps the key to unravelling the role of PRL in lupus is determining the source, given that lymphocytes, skin and hair follicles are all extra-pituitary sources [12,48,83,119,120,121]. This is all the more important when considering that PRL gene expression is regulated in a complex temporal and time-specific manner [122]. Furthermore, there is evidence from the skin and hair follicle that PRL exerts site- and sex-specific effects in peripheral tissue, likely in an autocrine and/or paracrine manner and independent of its hormonal effects [74,123,124]. Indeed, targeting local PRL expression, potentially via topical application of a PRLR antagonist [125], could represent an elegant treatment option for skin manifestations of lupus. Topical application of a PRLR antagonist would allow signalling to be modulated without affecting circulating PRL concentrations; this is particularly desirable given that PRL have over 300 biological effects on processes ranging from reproduction, lactation, angiogenesis, and immunomodulation through to osmo- and thermoregulation [126].

### 2.6. PRL and Psoriasis

Although the advent of biologic therapy has transformed the modern-day management of psoriasis, a common, chronic T-cell-mediated disease, it is still associated with significant physical and psychological comorbidity. There is extensive literature reporting an association between psychological stress and triggering and/or exacerbation of psoriasis, which has recently been the focus of systemic reviews and one meta-analysis [127,128,129,130,131,132]. Whether psychological stress can actually trigger psoriasis and the extent to which it can exacerbate pre-existing disease cannot be firmly concluded from the evidence to date. As Snast et al. [130] reported, most of the available evidence came from cross-sectional and retrospective studies, which are unsuited to determining cause and effects. One cohort study revealed a modest effect of stress on disease onset and exacerbation [133]. Nonetheless, given that cognitive behaviour therapy and behavioural interventions have been shown to decrease depression, anxiety, and psoriasis severity [134], an intimate connection between the brain and the skin is difficult to refute.

Much of the existing literature points towards the involvement of interleukin 6 (IL-6) along the brain–skin axis [128,135,136,137]. In fact, both the IL-6 receptor and PRLR belong to the same cytokine receptor superfamily due to their structural homology [138]. Thus, given PRL’s role as a stress hormone [128,139], it was reasonable to question whether PRL was a potential mediator of the effect of stress on psoriasis. In fact, bromocriptine had already been shown to induce resolution of psoriatic plaques in up to 70% of patients treated, albeit in a small, non-placebo-controlled trial [140].

Similar to that seen in lupus, elevated serum and/or cutaneous PRL levels were reported patients with psoriasis in several small studies, but not universally replicated [20,40,141,142,143,144]. Psoriasis was also reported in association with a prolactinoma in a case series [145]. Ultimately a meta-analysis was required to conclusively answer whether serum PRL was elevated in psoriasis. Indeed, not only did Lee et al. [41] confirm that circulating PRL levels are higher in patients with psoriasis, but they also confirmed that PRL levels may correlate with psoriasis severity. This is consistent with the evidence from several studies that both local and systemic psoriasis treatments can reduce circulating PRL levels, correlating with treatment response [146,147,148,149,150]. Most recently, PRLR expression in the skin has been confirmed in lesional and non-lesional skin in patients with psoriasis [39] and PRL expression is significantly expressed in lesional skin [40]. The difference in expression between lesional and non-lesional skin is particularly interesting, since it argues against cutaneous expression simply reflecting serum PRL concentrations and provides more evidence for increased intracutaneous PRL production and expression.

In addition, circulating PRL in general, and skin-derived PRL in particular, may influence several key facets of psoriasis pathology. For example, PRL stimulates keratinocyte proliferation [151], can promote angiogenesis [152], may enhance interferon gamma (IFN)-induced CXCL9, CXCL10, and CXCL11 production in keratinocytes, potentially promoting type 1 T cell infiltration into psoriatic lesions [153], and is associated with increases in Th1 and Th17 cytokine production in humans and in murine models of psoriasis [21,154]. Moreover, the dopamine agonist cabergoline could ameliorate skin lesions in the imiquimod-induced psoriasis-like mouse model [21].

Future studies of the role of PRL in psoriasis need to simultaneously examine the effects and extent of circulating and intra-cutaneous production in addition to rigorously measuring stress, ideally in a suitably powered prospective study. Given the acute changes in PRL expression following stress exposure, the Trier Social Stress Test may offer a laboratory-controlled, standardised method to evaluate the role of PRL in the cutaneous response to stress and to determine whether this is dysregulated in patients with psoriasis.

### 2.7. PRL and Wound Healing

The management of chronic leg ulcers and wound healing disorders represents a major medical challenge, and both are associated with significant morbidity and quality-of-life impairments [155]. Given that PRL plays a regulatory role in many of the key processes involving in wound healing, including keratinocyte proliferation, angiogenesis, hair follicle cycling, and stem cell function, it is certainly feasible that PRL could modulate cutaneous wound healing.

Several PRL fragments have now been identified which are involved in the response to tissue injury. For example, vasoinhibin (both 14 kDa and 5.6 kDa fragments) has potent pro-fibrinolytic, anti-vasopermeability, and anti-angiogenic effects [156]. Thrombin, plasmin, cathepsin D, and matrix metalloproteinases have all been shown to cleave 23kDa PRL into variants which are involved in wound healing and tissue repair [22,156,157,158]. PRL has been shown to be a negative regulator of female human skin repair in ex vivo skin organ culture [47]. When added to wounded skin ex vivo, 23kDa PRL inhibited epidermal re-epithelialisation and promoted keratinocyte terminal differentiation whilst simultaneously inhibiting cytokeratin 6 protein expression and intra-epidermal mitochondrial activity (MTCO1 expression).

In contrast, recent evidence from rodent studies has suggested that PRL may promote wound healing in vocal fold injuries, primarily based on decreased PRL expression following injury [159]. The authors pointed out that a reduction in PRL expression does not necessarily mean that increased expression promotes wound healing. Clinical data examining the relationship between PRL and cutaneous wound healing are sparse, although one case of impaired wound healing following mammoplasty, accompanied by hyperprolactinaemia, has been reported [160] in addition to a case of hypertrophic breast scarring and hyperprolactinaemia in a patient with burn injuries [161].

A comprehensive evaluation of the relationship between PRL and cutaneous wound healing in vivo is currently lacking. Nevertheless, based on the evidence to date, and given the site- and sex-dependent effects of PRL on human hair growth [123], the effects of PRL and PRL variants on wound healing deserve further attention.

### 2.8. PRL and the Sebaceous Gland

Returning to the pilosebaceous unit, where seborrhoea was reported in patients with Parkinson’s Disease over half a century ago and reportedly responded to treatment with levodopa [17,72,162], the role of PRL in sebaceous gland biology has received little attention. This is perhaps surprising, given that the most common disease affecting the pilosebaceous unit, acne vulgaris, is often exacerbated by psychological stress and associated with psychological morbidity [163,164,165]. Acne is recognised in association with hyperprolactinaemia in females, possibly resulting from increased dehydroepiandrosterone sulphate secretion from the adrenal glands, which results in clinical signs of hyperandrogenism [166]. Hyperprolactinaemia has been reported in up to 45% of females with persistent and/or late-onset acne [167], albeit based on small studies, and associated with stress in patients with acne [168]. Until recently, any effects of PRL on the sebaceous gland were thought to be modulated exclusively via its effects on androgen metabolism [169,170,171,172]. Isotretinoin, a mainstay of treatment for severe acne, has also been shown to decrease serum PRL concentrations [173,174]. Bromocriptine and dopamine agonists are also reportedly affected in the management of acne [169,175].

Clinically, a diagnosis of hyperprolactinaemia can only be made when serum PRL levels are elevated on two separate occasions [166] and a careful diagnostic work-up is required, given that the cause may be broadly categorised as physiological (including stress and exercise), pharmacological (dopamine receptor antagonists, oral contraceptive pill), or pathological (prolactinoma, liver cirrhosis, polycystic ovarian syndrome, chest wall trauma, and seizures). The list of causes of hyperprolactinaemia is therefore extensive. Nevertheless, PRL is often included in an androgen work-up in patients with acne, hirsutism, and menstrual irregularities and obesity [176].

The expression of PRL receptors in the sebaceous gland provides more direct evidence that PRL is a sebotrop(h)ic hormone [83,119,120]. Moreover, PRL increases sebaceous gland size, sebocyte proliferation, lipid production, and 5 alpha reductase type I expression when added to female skin organ culture ex vivo [75]. That latter observation is particularly interesting, since it suggests that PRL can locally stimulate androgen metabolism, which is of potential significance not only in acne but also in androgenetic hair loss.

### 2.9. Future Directions

A combination of standardised laboratory stress tests in humans measuring central and peripheral PRL expression, potentially complemented by ex vivo skin and hair follicle organ culture experiments, may provide the best way forward. Moreover, the generation of commercially available PRLR antagonists could be a game changer in terms of allowing PRLR signalling to be targeted therapeutically to manage stress-mediated skin diseases. What is currently clear is that although PRL plays a crucial role in mammalian biology, targeting a modified sweat gland to regulate lactation, we have barely begun to understand its role in the skin.

## 3. Conclusions

Over the past decade, there has been significant progress in our understanding of PRL as a prototypical stress hormone in the cutaneous context. Based on its regulatory role in cutaneous immunity, angiogenesis, keratinocyte and stem cell biology, hair follicle growth and cycling, sebaceous gland function, and androgen metabolism, it is not surprising that PRL is involved in diseases ranging from acne and autoimmune blistering diseases to psoriasis and urticaria [47,159]. The challenge facing PRL research in the next decade will be uncovering the extent to which acute and chronic stress can mediate intracutaneous PRL expression and unrevealing the effect of pituitary and extra-pituitary-derived PRL on the skin and cutaneous immune system. A combination of standardised laboratory stress tests in humans, measuring central and peripheral PRL expression, potentially complemented by ex vivo skin and hair follicle organ culture experiments, may provide the best way forward. Moreover, the generation of commercially available PRLR antagonists could be a game changer in terms of allowing PRLR signalling to be targeted therapeutically to manage stress-mediated skin diseases. What is currently clear is that although PRL plays a crucial role in mammalian biology, targeting a modified sweat gland to regulate lactation, we have barely begun to understand its role in the skin.

## Figures and Tables

**Table 1:** Skin disease and hair disorders associated with prolactin.

Skin Disease	Sex	Findings	Reference
Acne	F	Elevated PRL concentrations in 3.3% of cases of adult female acne No association with acne severity	[23]
	M/F	Elevated PRL concentrations in patients with acne (males and females) compared to age- and BMI-matched controls	[24]
	F	No elevated PRL concentrations in females with late-onset or persistent acne	[25]
	F	Low-dose isotretinoin significantly reduced serum PRL concentrations	[26]
Alopecia	F	Case report of frontotemporal hair loss in association with hyperprolactinaemia in a patient with autoimmune thyroid disease	[27]
Alopecia areata (AA)	M/F	No difference in serum PRL concentrations between patients and healthy controls, but increased PRL receptor expression in scalp biopsies of patients with AA, which correlated with disease severity	[28]
Atopic Dermatitis (AD)	M/F	No increased PRL serum concentrations in patients with AD compared to controls and no correlation with disease severity	[29]
Frontal fibrosing alopecia	F	No difference in serum PRL concentrations between patients and age- and menopause status-matched controls	[30]
Female pattern hair loss	F	Measure of serum PRL optional but recommended based on the report from the multidisciplinary androgen excess and polycystic ovary syndrome (PCOS) committee	[31]
	F	Hyperprolactinaemia identified in 7.2% of patients	[32]
	F	Moderate elevated serum PRL concentration unlikely to play a causative role in female hair loss	[33]
Lichen Planus (LP)	M/F	No difference between PRL serum concentrations in patients with oral LP and aged- and sex matched controls	[34]
Pemphigus	M/F	Elevated PRL concentrations in 20% of patients newly diagnosed with pemphigus vulgaris Correlation with severity (Pemphigus Disease and Severity Index)	[35]
	F	Elevated PRL concentrations in 22% of patients newly diagnosed with pemphigus vulgaris No correlation with clinical or serological disease activity	[36]
	M/F	Elevated PRL concentrations in patients newly diagnosed with pemphigus vulgaris compared to age- and sex-matched controls Significant association between severity of pemphigus and serum PRL concentrations	[37]
	M/F	No correlation between serum PRL concentration and anti-desmoglein 1 or 3 levels.	[38]
Psoriasis	M/F	PRL Receptor immunoreactivity in lesional- and perilesional skin in the sweat glands and hair follicle outer root sheath	[39]
	M	Prolactin level is significantly elevated in lesional skin of patients with psoriasis	[40]
	M/F	Meta-analysis confirms increased serum PRL concentrations in patients with psoriasis, which may correlate with disease severity	[41]
	M/F	Elevated serum PRL concentrations in males and females and lesional PRL concentrations correlated with disease severity	[21]
	M/F	Elevated serum PRL concentrations in patients with psoriatic arthritis when compared to patients with psoriasis and healthy controls	[42]
	M/F	No difference in serum PRL concentrations between patients with psoriasis, AD, and healthy controls	[20]
Melanoma	M	Case report of hyperprolactinaemia in metastatic melanoma, possibly via ectopic production, normalised during immune checkpoint inhibitor therapy with pembrolizumab	[43]
Systemic sclerosis (SSc)	M/F	Meta-analysis revealing significantly higher PRL concentrations in SSc patients than healthy controls; sex- and detection method-dependent	[44]
Urticaria	F	20% of the patients with chronic urticaria had elevated serum PRL concentrations and 50% had a clinical remission with bromocriptine treatment	[45,46]
Wound healing	F	PRL significantly inhibited epidermal regeneration (reepithelialisation), cytokeratin 6 protein expression, and intraepidermal mitochondrial activity (MTCO1 expression), while it promoted keratinocyte terminal differentiation (i.e., involucrin expression) ex vivo.	[47]

**Table 2:** Factors influencing serum PRL concentrations.

Factors Influencing Serum PRL Concentrations [59,60,61]	Examples
Physiological factors	Sex
	Age
	Diurnal variation
	Psychological stress
	Pregnancy, lactation
	Nipple Stimulation
	Menopausal status
	Exercise
Pathological factors	Tumours—for example, prolactinoma
	Hypothalamic diseases, e.g., Sarcoidosis
	Pituitary stalk disorders, e.g., Tuberculosis, Langerhans cell Histiocytosis
	Trauma, including brain or chest wall injury
	Genetic—PRL receptor (PRLR) mutation
	Hypothyroidism
	Chronic renal failure
	Ectopic production
Medication	Antidepressants
	Antiemetics
	Antipsychotics
	Antihypertensives
	Opioids
	Oral contraceptives
Idiopathic	No underlying cause found
